# Epidemiological trends in psoriatic arthritis: a comprehensive population-based study

**DOI:** 10.1186/s13075-024-03339-0

**Published:** 2024-05-27

**Authors:** Amir Haddad, Perach Chen Elkayam, Nili Stein, Ilan Feldhamer, Arnon Dov Cohen, Walid Saliba, Devy Zisman

**Affiliations:** 1 Rheumatology Unit, Carmel Medical Center, 7 Michal Street, Haifa, Israel; 2 Department of Epidemiology, Clalit health services, Haifa, Israel; 3 Chief Physician’s Office, Central Headquarters, Clalit Health Services, Tel Aviv, Israel; 4 Siaal Research Center for Family Medicine and Primary Care, Faculty of Health Sciences, Ben- Gurion University of the Negev, Beer-Sheba, Israel; 5 Ruth and Bruce Rappaport Faculty of Medicine, Technion-Israel Institute of Technology, Haifa, Israel

**Keywords:** Psoriasis, Psoriatic arthritis, Spondyloarthritis, Epidemiology, Middle East

## Abstract

**Background:**

Psoriatic arthritis (PsA) is a chronic, potentially debilitating inflammatory arthritis often associated with psoriasis. Understanding the epidemiology of PsA across diverse populations can provide valuable insights into its global burden and the role of genetic and environmental factors. This study aimed to estimate PsA’s temporal trends, prevalence, and incidence, while assessing variations in age, gender, and ethnicity in Israel from 2016 to 2022.

**Methods:**

Data were sourced from the Clalit Health Services (CHS) database, covering over half of the Israeli population. Algorithm-based definitions for PsA and psoriasis cases were used. Demographic factors, including age, gender, socioeconomic status (SES), ethnicity, urban/rural residence, BMI, and smoking status, were analyzed. Standardized prevalence and incidence rates were calculated. Logistic regression analyses examined associations of sociodemographic variables with PsA.

**Results:**

In 2022, the prevalence of PsA was 0.221%, with an incidence rate of 13.54 per 100,000 population. This prevalence has tripled since 2006, reflecting a rising trend in PsA over time. Females exhibited a higher prevalence (1.15; 95%CI 1.09–1.21), and PsA was more common in Jewish individuals (1.58; 95%CI 1.45–1.71) those with higher SES (1.4; 95% CI 1.31, 1.5), and those with obesity (2.17; 95%CI 2.04–2.31).

**Conclusions:**

This comprehensive population-based study pointed to an increase prevalence of PsA, emphasizing the rising healthcare demands and economic burden faced by this patient population. Further research is essential to delve into the factors driving these trends.

## Background

Psoriatic arthritis (PsA) is a chronic, potentially destructive inflammatory arthritis that is associated with comorbidities and affects individuals with psoriasis.

Investigating PsA’s epidemiology in diverse populations holds the potential to enhance our comprehension of the global disease burden. Moreover, given the significant influence of environmental and genetic factors on PsA susceptibility, exploring this condition’s epidemiology in ethnically varied populations across different geographic regions could shed light on the underlying mechanisms of the disease.

The reported prevalence of PsA worldwide ranges from 0.1 to 1% in the general population [1, 2].

A 2018 systematic review and meta-analysis identified a PsA prevalence of 133 (95% CI, 107–164) per 100,000 individuals (or 0.13%) within the general population [3]. Considerable heterogeneity among the included studies was observed in the review, likely stemming from variations in geographic location, target demographics, research methodologies, genetic backgrounds, environmental and lifestyle factors. Additionally, differences in PsA case definitions contributed to the observed heterogeneity.

Most investigations into PsA prevalence trends indicate a rise in prevalence in recent years, whereas fewer studies addressing incidence trends over time yield inconsistent findings [4].

Scant data exists regarding PsA’s epidemiology in Middle Eastern populations representing various ethnic backgrounds [5, 6]. In a prior study conducted by our group, the prevalence of PsA in the adult Israeli population in 2015 was 0.153%. This marked a doubling of the prevalence in this population over the preceding decade, while the incidence remained stable during the same period [7]. As the prevalence is a function of incidence and disease duration, this increase suggests a possibility of a genuine increase in disease duration that might be as a consequence of a combination of population aging or growth and improvement in disease treatment or a decrease in mortality over the years as was observed in a previous study on this population[8].

The objectives of our study encompassed estimating the temporal trends in PsA prevalence and incidence, as well as assessing variations among different age, gender, and ethnic subgroups in the general population of Israel spanning from 2016 to 2022.

## Methods

### Patient and data source

The study utilized data spanning from 2016 to 2022, sourced from the Clalit Health Services (CHS) database. CHS is one of the four nonprofit health organizations delivering both hospital-based and community-based healthcare services, encompassing medical treatments, diagnostic tests, and hospitalizations in the Israeli population. Israeli law mandates all residents to choose coverage with one of these health maintenance organizations, with CHS being the largest, serving approximately 4.7 million enrolees, constituting 52% of Israel’s total population. The demographic makeup, geographic distribution, and ethnic diversity of the population served by CHS closely resemble that of the broader Israeli population. The CHS database comprises comprehensive demographics and clinical data, incorporating sociodemographic information derived from Israel’s Central Bureau of Statistics and the National Insurance Institute (Social Security). The database is continually updated with information from pharmaceutical, medical, and administrative systems. It consolidates data from electronic medical records originating from primary care and specialist clinics, hospitals, pharmacies, and laboratories. A registry of chronic diseases diagnoses is compiled from these data sources. Diagnoses are captured in the registry by diagnosis-specific algorithms, employing International Classification of Diseases Ninth revision (ICD-9) code reading, laboratory test results and disease-specific drug usage. A record is kept of the data-sources and dates used to establish the diagnosis, with the earliest recorded date, from any source, considered to be the defining date of diagnosis. The CHS database has been instrumental in various epidemiological studies, including those in the field of psoriatic disease.

### Study population

The study encompassed all individuals aged 18 years and above who were enrolled in CHS at any point between 2016 and 2022. Patients were tracked from their enrolment with CHS until their demise, departure from CHS, or the conclusion of the study in December 2022. The primary analysis pertaining to the global and subgroup prevalence of PsA involved the entire CHS population in 2022. Temporal trends in the prevalence and incidence rates of PsA from 2016 to 2022 were evaluated. For this analysis, the study population comprised all CHS members aged 18 years and older for each respective year. All data utilized in this study were rendered anonymous to the researchers. The study received approval from the research ethics board of Carmel Medical Centre.

### Psoriatic arthritis and psoriasis case definition

A pilot study was conducted to validate an algorithm for identifying patients with PsA within the CHS database[9]. Briefly an algorithm that encompassed the following conditions was applied: (1) PsA diagnosis assigned by a rheumatologist at least once; (2) a permanent diagnosis code assigned by a primary care physician, combined with the use of synthetic or biologic disease-modifying antirheumatic drugs; or (3) a PsA code listed in a hospitalization discharge summary. This algorithm exhibited a positive predictive value, sensitivity, and specificity of 90.5%, 88.7%, and 88.1%, respectively.

The process for identifying patients with psoriasis in the CHS database has been detailed in earlier publications[10, 11]. In essence, individuals were classified as having psoriasis if their medical records contained at least one documented diagnosis of psoriasis by a CHS dermatologist or if psoriasis was included in the diagnosis section of hospital discharge letters.

### Additional information

The following variables were extracted from the database: age, gender, socioeconomic status (SES), ethnicity (Jewish or Arab), area of residence (urban vs. rural), body mass index (BMI), and smoking status. SES was categorized as low, middle, or high based on neighborhood socioeconomic scores established by the Israel Central Bureau of Statistics, representing a Z-score comparing the mean SES of the subject’s neighborhood with that of the overall neighborhood[12].

### Statistical analysis

Crude and age- and sex-standardized annual prevalence and incidence rates (with corresponding 95% confidence intervals) for PsA were computed by dividing the number of PsA patients (aged 18 and over) by the total count of CHS enrollees (aged 18 and over) for the respective year within the 2016–2022 period. Disease onset was defined as the date of the first qualified health services contact for which a PsA diagnosis was documented. Patients with such initial contacts were considered incident PsA cases. Prevalent cases were carried forward from the date of their first PsA diagnosis until they met one of the following endpoints: death, departure from CHS, or the study’s conclusion in December 2022. Annual sex- and age-specific prevalence and incidence rates were determined for 10-year age groups and expressed as proportions (95% confidence intervals) using the 2006 Israeli population for direct age and sex standardization[13] so we could compare the numbers to our previous study that was conducted over the years 2006–2015. Furthermore, the crude prevalence of PsA (in 2022) was separately reported for the following subgroups: gender (males vs. females), ethnicity (Jewish vs. Arab), SES (high, moderate, low), area of residence (urban vs. rural), BMI category (underweight, normal, overweight, obese), and smoking status (smokers vs. non-smokers). Information on model covariates was drawn from the CHS database for the year 2022. Results were presented as odds ratios with 95% confidence intervals.

## Results

Among the 3,133,500 individuals aged 18 years and older registered in the CHS database in 2022, 6930 patients had a diagnosis of PsA; of those, 432 patients had a new diagnosis of PsA, resulting in an overall crude prevalence rate of 0.221% and incidence rate of 13.9 per 100,000 population (Table 1). Additionally, 42,046 patients had a diagnosis of psoriasis, resulting in an overall crude prevalence of 1.34% (95% CI 1.327%, 1.353%). The prevalence of PsA among patients with psoriasis (in 2022) was 16.48%.

**Table 1:** Prevalence and incidence of psoriatic arthritis in Israel by age and sex in 2022

Age Group	Male			Female			All			
	PSA (*n*)	Total (*N*)	Prevalence (%)	PSA (*n*)	Total (*N*)	Prevalence (%)	PSA (*n*)	Total (*N*)	Prevalence (%)	Incidence per 100,000
18–29	80	364,672	0.022	155	374,549	0.041	235	739,221	0.032	6.418
31–39	345	313,226	0.110	399	321,058	0.124	744	634,284	0.117	15.66
41–49	604	272,858	0221	616	269,839	0.228	1220	542,697	0.224	16.67
51–59	647	186,832	0.346	756	199,179	0.380	1403	386,011	0.363	21.68
61–69	746	178,791	0.417	950	206,312	0.460	1696	385,103	0.440	18.92
71–79	546	129,505	0.421	689	157,910	0.436	1235	287,415	0.430	11.45
81+	165	61,431	0.268	232	97,338	0.238	397	158,769	0.250	5.56
All	3133	1,507,315	0.208	3797	1,626,185	0.233	6930	3,133,500	0.221	13.90

**Fig. 1:**
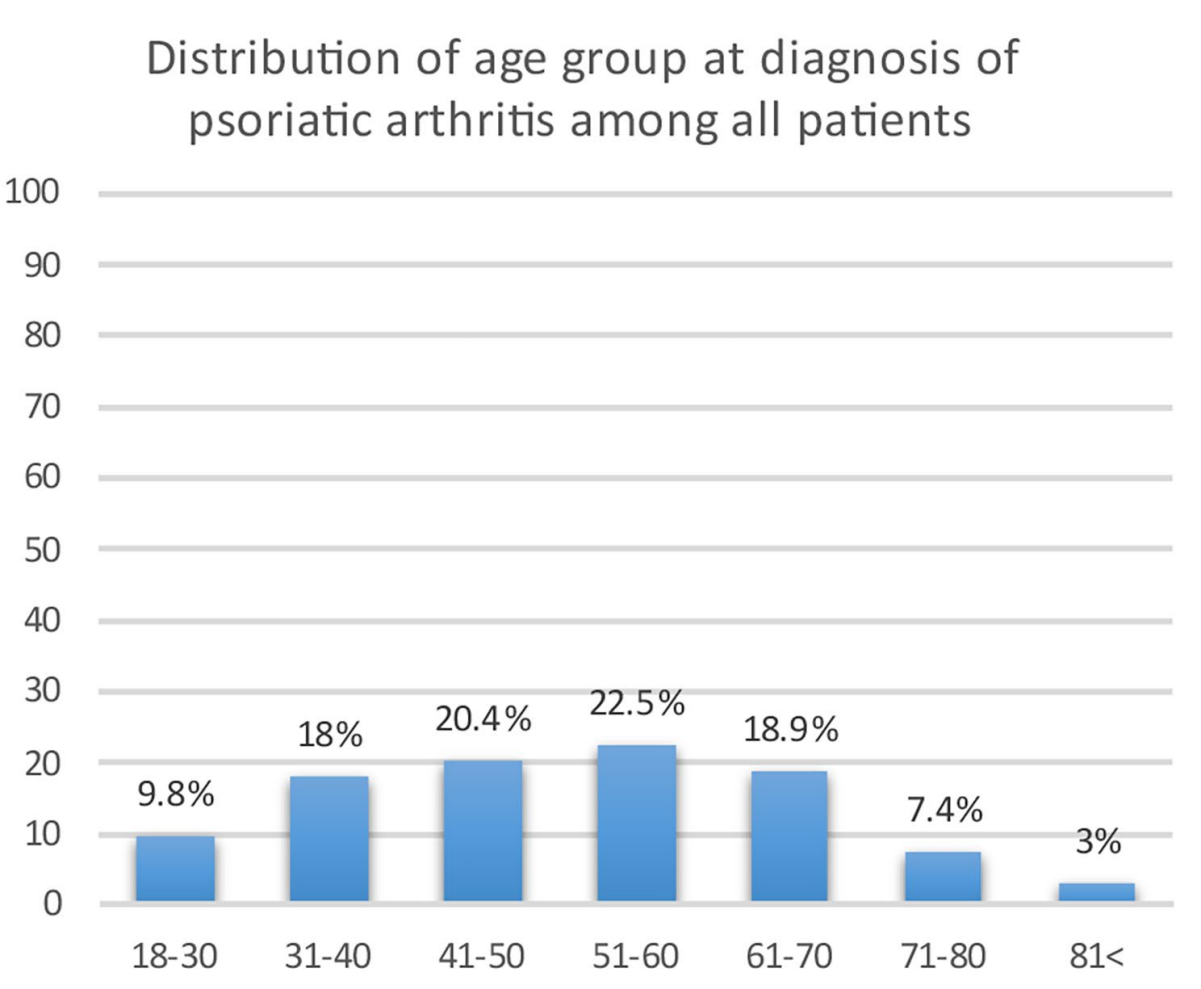
The distribution of age at diagnosis of psoriatic arthritis among all patients

The prevalence of PsA was slightly higher in females (0.233%) than in males (0.208%). The prevalence of PsA peaked in the sixth decade of life, at 0.440%. The age at diagnosis of PsA was higher than reported in previous cohorts from Europe and North America, since over half the patients were diagnosed after the age of 50 years (Fig. 1).

Most patients with PsA had a recorded diagnosis of psoriasis (5671 patients [81.4%]). In the majority (3363 [59.3%]), the diagnosis of PsA was recorded following the diagnosis of psoriasis. In 1925 patients (33.95%), the diagnoses of PsA and psoriasis were recorded in the same year, and in the remaining 6.75% (383) of patients, the diagnosis of PsA preceded the diagnosis of psoriasis.

### Temporal trend in the prevalence and incidence of PsA from 2016 to 2022

The crude prevalence of PsA has increased during the study period, from 0.165% in 2016 to 0.221% in 2022 (Table 2) and compared to 2006, it had tripled (the crude prevalence rate at that time was 0.073% as reported in a previous study). A similar increase was observed in the age- and sex-standardized PsA prevalence: from 0.148% in 2016 to 0.197% in 2022.

**Fig. 2:**
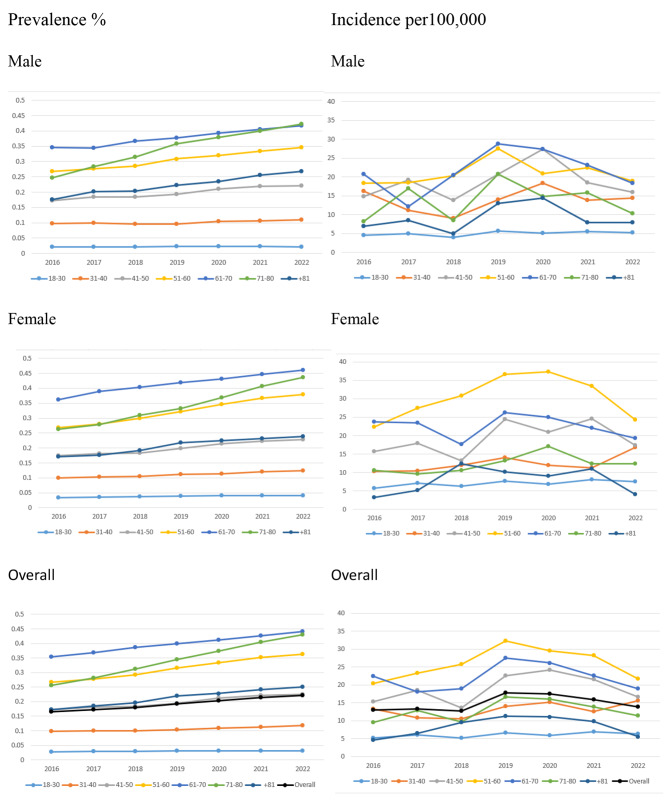
Prevalence and incidence of psoriatic arthritis in Israel by age and sex group from 2016–2022

There was also a trend on increase in the crude incidence rate over the years 2016–2022, as it has increased from 12.98 up to the range of 17.75 and 17.51 in the years 2019 and 2020 respectively, then gradually decreased to 13.90 per 100,000 population in 2022 as well as an increasing trend in the the age- and sex-standardized incidence rates from 12.4 to 13.54%. The prevalence and incidence rates increased in both sexes over time. The temporal trends in sex-standardized prevalence and incidence of PsA by age groups are shown in Fig. 2. A more pronounced increase in the prevalence of PsA was observed in the age group (51–60 years) in females.

**Table 2:** Crude and age- and sex-standardized prevalence and incidence of psoriatic arthritis in Israel by year (2016–2022)

	Prevalence of PsA				Incidence of PsA		
Year	Total population	PSA cases (*n*)	Crude prevalence (%)	Standardized prevalence (95% CI)	New PSA (*n*)	Crude incidence	Standardized incidence ( 95% CI)
2016	2,898,800	4,782	0.165	0.148 (0.146–1.15)	374	12.98	12.40 (11.1–13.7)
2017	2,934,164	5,081	0.173	0.155 (0.153–0.157)	389	13.36	13.05 (11.7–14.4)
2018	2,974,138	5,372	0.181	0.161 (0.159–0.163)	374	12.66	12.25 (11-13.5)
2019	3,015,102	5,807	0.193	0.172 (0.17–0.174)	531	17.75	17.07 (15.6–18.5)
2020	3,057,362	6,225	0.204	0.182 (0.18–0.184)	532	17.51	16.74 (15.3–18.2)
2021	3,092,199	6,602	0.214	0.190 (0.188–0.193)	488	15.90	15.46 (14-16.8)
2022	3,133,500	6,930	0.221	0.197 (0.194-0.2)	432	13.90	13.54 (12.2–14.8)

### Factors associated with PsA

The prevalence of PsA was associated with several demographic and lifestyle factors, as shown in Table 3. We found an association of female sex and PsA. PsA was more frequent in female individuals (prevalence 0.233%, adjusted OR of 1.15; 95% CI 1.09–1.21). An association was found between the level of SES and PsA. PsA was most frequent in people with a higher SES (high SES prevalence 0.286%, adjusted OR high vs. low SES (1.4; 95% CI 1.31, 1.5), followed by middle SES (moderate SES prevalence 0.243%, OR middle vs. low SES 1.17; 95% CI 1.10, 1.24) and lowest in the low SES (prevalence 0.169%). Additionally, the distribution of PsA varied by ethnicity, with higher prevalence in individuals of Jewish ethnicity compared with Arabs (adjusted OR 1.58, 95% CI 1.45–1.71). Moreover, PsA was more frequent in overweight (prevalence 0.277%; OR 1.66; 95% CI 1.56–1.77) and obese individuals (prevalence 0.353%; OR 2.17; 95% CI 2.04–2.31) than in people with normal weight (prevalence 0.170%). Lastly, smoking was also associated with PsA (prevalence 0.283%, adjusted OR of 1.42; 95% CI 1.35–1.49).

**Table 3:** Prevalence of psoriatic arthritis by demographic characteristics (in 2022) and their association with psoriatic arthritis by logistic regression analysis

	Prevalence (%)	Univariate model OR. 95% CI	Multivariate model OR, 95% CI
Sex			
Female	0.233%	1.12; 95% CI 1.07–1.18	1.15; 95% CI 1.09–1.21
Male	0.208%	Ref.	Ref.
Socioeconomic status			
High	0.286%	1.70; 95% CI 1.59–1.80	1.4; 95% CI 1.31, 1.5
Middle	0.243%	1.44; 95% CI 1.37–1.53	1.17; 95% CI 1.10, 1.24
Low	0.169%	Ref.	Ref.
Ethnicity			
Jewish	0.252%	1.90; 95% CI 1.78–2.02	1.58; 95% CI 1.45–1.71
Arab	0.139%	Ref.	Ref.
Residential area			
Urban	0.240%	1.29; 95% CI 1.22–1.36	0.99; 95% CI 0.94–1.05
Rural	0.187%	Ref.	Ref.
BMI, kg/m^2^			
>30	0.353%	2.08; 95% CI 1.95–2.21	2.17; 95% CI 2.04–2.31
25–30	0.277%	1.63; 95% CI 1.53–1.73	1.66; 95% CI 1.56–1.77
18.5–25	0.170%	Ref.	Ref.
<18.5	0.122%	0.72; 95% CI 0.58–0.89	0.71; 95% CI 0.58–0.88
Smoking			
Yes	0.283%	1.52; 95% CI 1.47–1.59	1.42; 95% CI 1.35–1.49)
No	0.187%	Ref.	Ref.

## Discussion

In this population-based study, we examined the the temporal trends in the prevalence and incidence of PsA in a large datsbase in Israel from 2016 to 2022. The age and sex standardized prevalence and incidence rates were calculated using the 2006 Israeli population to allow a direct comparison to the rates provided in the previous study. We observed that in 2022, the prevalence of PsA in the adult population in Israel was 0.221% with an incidence rate of 13.54 per 100,000 population. The reported prevalence of PsA in Israel has tripled since 2006, rising from 0.067% in 2006 to 0.221% in 2022. The global incidence rate has also relatively increased from 10.4 (95% CI 9.5–11.4) in 2006 to 15.5 (95%CI 14-16.8) in 2021 and 13.5 (95%CI 12.2–14.8) in 2022 and that PsA was more commonly found in individuals from specific groups, including females, Jewish ethnicity, higher SES, higher BMI and among smokers.

Most population-based epidemiological studies on PsA have focused on European populations, leaving a significant gap in our understanding the disease prevalence in other ethnic groups and geographic regions, particularly in Middle Eastern populations.

To the best our knowledge, this study stands as the largest-scale population-based research providing insights into PsA prevalence in a Middle Eastern population, encompassing two diverse subpopulations.

Our estimated PsA prevalence in Israel (0.221%) falls above the prevalence range reported in a systematic review and meta-analysis (0.13%) the general population [4], but aligns more closely with estimates in the United States (0.25%)[14], Ontario[15] and Northern European countries such as Sweden (0.25%)[16], Norway (0.19–0.67%)[17, 18], the United Kingdom (0.19%)[5].

Discrepancies in prevalence estimates between studies are attributed to factors like differences in study design, case definitions (self-reported or database-derived), genetic backgrounds, environmental factors (including climate and infections), lifestyle (smoking, alcohol consumption, and obesity), and dietary habits (such as adherence to the Mediterranean diet and fish oil consumption).

Notably, the prevalence of PsA varies in other Mediterranean countries, ranging from 0.05% in Turkey[19], to 0.06–0.17% in Greece[20, 21].

Fewer studies have explored the incidence of PsA. Our estimated incidence rate of cases per 100,000 population falls within the range of previous estimates in most European and US populations, which typically range from 6 to 35.9 per 100,000 [4].

Traditionally, the proportion of male and female PsA patients has been considered roughly equal. However, slight variations in sex proportions have been reported in different studies. [4] In our study we observed a slight female predominance (0.233% vs. 0.209%) and both sexes exhibited an increasing incidence over time.

The prevalence of PsA among patients with psoriasis in our study was 16.48%, compared to a pooled PsA prevalence of 19.7% (95% CI 18.5–20.9%) in patients with psoriasis in a 2019 systematic review and meta-analysis.[22].

Our study demonstrates an increasing trend in the crude and age-adjusted prevalence and incidence of PsA over the study period. These findings align with recent research from Europe and Asia that has reported a rise in the prevalence and incidence of psoriasis and PsA over time[23–25]. Possible factors contributing to this trend include increased disease awareness among physicians, possibly driven by the 2006 Classification Criteria for Psoriatic Arthritis (CASPAR) criteria, which heighlighted awarness and recognition of PsA and might have lead more rheumatologists to classify a disease as “PsA” rather than “spondyloarthritis”. Morever, the enhanced and increased utilization of use of an advaced and sensitive diagnostic modalities (e.g., ultrasound and magnetic resonance imaging- MRI) could have impacted PsA diagnosis. Additionally, both international and local educational initiatives among rheumatologist and primary care physicians as well as rheumatologist and dermatology specialists (Group for Research and Assessment of Psoriasis and psoriatic arthritis **-**GRAPPA) has led to an increase in disease awareness and more referral to a rheumatologist.

Other potential drivers for the rise in PsA prevalence could be the decrease in disease mortality over the past decades, that could be the as a result of improvement in disease management and treatments over the years. Additionally, the increased presence of known risk factors like obesity in Israel [26, 27] and shifts in population demographics such as immigration of high-risk groups could have also impacted these trends.

Moreover, greater availability of effective medications can also lead to more individuals seeking medical advice and consequently being diagnosed with the disease.

Various demographic factors were found to be correlated with the occurrence of PsA including sex, ethnicity, SES, BMI and smoking status. The prevalence of PsA was higher in females, consistent with studies that showed higher incidence of PsA in females[22, 28]. Nevertheless, the data on sex ratio also appears disparate. PsA was also nearly 1.6 times more prevalent in the Jewish population compared to Arabs, as well as among individual with higher SES. These disparities may be attributed to differences in genetics, environmental exposures, or healthcare utilization.

Furthermore, our findings confirm the association of obesity with a nearly twofold increase in PsA prevalence, aligning with the existing literature. [29, 30]

Additionally, our study suggests an association of smoking with PsA (adjusted OR of 1.42; 95% CI 1.35–1.49). However, these results should be interpreted with cation as we didn’t account for psoriasis as a causal intermediate variable. Thus, smoking might indirectly elevate the risk of PsA by increasing the risk of psoriasis, potentially leading to spurious findings. Smoking is well-known risk factor for psoriasis; yet its link to the development of PsA has produced varying results in prior research [31, 32].

Our study carries several limitations. We could not capture patients with PsA or psoriasis who did not seek medical attention or those who remained undiagnosed. Additionally, our case definition relies on diagnostic coding from electronic medical records based on physician diagnoses, which carries the risk of misclassification. Nonetheless, we minimized this risk by employing an algorithm with high accuracy that largely relied on specialist diagnoses.

A significant strength of our study is the use of a large, representative sample covering over half the Israeli population, allowing for broad generalization of our findings.

## Conclusions

High-quality real-world data concerning the epidemiology of PsA are essential for understanding the disease’s societal burden. Our study, involving approximately 7,000 PsA patients, stands as one of the most extensive population-based studies to date, assessing PsA prevalence in Middle Eastern individuals. Based on this large data base the prevalence of PsA in the adult Israeli population in 2022 was 0.221% and it tripled since 2006, signalling increasing healthcare demands and economic burden among this patient population. Further research is warranted to continue monitoring this trend and uncover its underlying determinants.

## Data Availability

All data in this study is available upon reasonable request from the corresponding author.

## Abbreviations

PsA: Psoriatic arthritis
CHS: Clalit health services
ICD-9: International Classification of Diseases Ninth revision
SES: Socioeconomic status
BMI: Body mass index
MRI: Magnetic resonance imaging
CASPAR: Classification Criteria for Psoriatic Arthritis
GRAPPA: Group for Research and Assessment of Psoriasis and psoriatic arthritis
